# X-Linked Lymphoproliferative Disease Presenting as Pancytopenia in a 10-Month-Old Boy

**DOI:** 10.1155/2010/517178

**Published:** 2010-06-21

**Authors:** S. Nicole Chadha, David Amrol

**Affiliations:** Departments of Pediatrics and Internal Medicine, University of South Carolina School of Medicine, 14 Medical Park Suite 400, Columbia, SC 29203, USA

## Abstract

X-linked lymphoproliferative disease, also known as Duncan's syndrome, is a rare genetic disorder that causes exaggerated immune responses to Epstein-Barr virus (EBV) infection and often leads to death. Patient presentation varies but can include signs and symptoms typical of EBV, pancytopenia, and fulminant hepatitis.

## 1. Case Report

A previously healthy 10-month-old boy was admitted with one week of fever, rash, and malaise. He appeared lethargic with a fever of 102 degrees F and a heart rate of 180 beats per minute. He had a purpuric petechial rash over his trunk and extremities and an erythematous bulging tympanic membrane on the right. His liver was palpable 4 cm below the costal margin and he had no splenomegaly. His initial white blood cell count was 3.9 with 68% lymphocytes and 13% atypical lymphocytes. His hemoglobin was 8.1 mg/dL and platelet count 36,000. His AST, ALT, and LDH were all elevated at 152, 146, and 2041, respectively. Epstein-Barr virus (EBV) serologies indicated acute infection and viral load demonstrated persistent viremia. His immunoglobulins were normal. 

He was treated with ceftriaxone for acute otitis media and possible bacteremia; over the next several days he improved clinically with less lethargy and improved appetite. However, his pancytopenia worsened necessitating red blood cell and platelet transfusions. His bilirubin rose to 8, ALT to 1890, and AST to 7300. His triglycerides were elevated at 319 and fibrinogen low at 107 (Table 1). A bone marrow biopsy revealed hypocellular marrow with no evidence of hemophagocytosis.

He was treated with high dose IVIG, acyclovir, steroids, and chemotherapy according to the HLH-94 protocol [1]. Three weeks into his illness he developed respiratory distress and was intubated. He developed progressive liver failure with coagulopathy and appeared septic. He died 24 days after the onset of nonspecific viral symptoms consistent with acute EBV infection.

Gene sequencing performed on a peripheral blood sample at the University of Washington identified a point mutation in exon 1 resulting in a new splice site and the deletion of 22 base pairs, frame shift, and early termination of SH2D1A, confirming a diagnosis of XLP. His mother is a carrier of the mutation. His soluble interleukin-2 (sIL-2R) receptor level was 9311 U/mL and perforin studies were normal. Natural killer cell function was not able to be performed.

X-linked lymphoproliferative disease (XLP) is a rare genetic disorder which affects less than one in one million people, usually previously healthy males in their first decade of life. The defective gene responsible for this disease is found on the X chromosome at Xq25 and encodes the protein SAP (signaling lymphocyte activation molecule or SLAM-associated protein, also called DSHP or SH2D1A), an important mediator of signal transduction in natural killer (NK) and T cells that ultimately lead to lymphocyte activation [2, 3]. More than 50 heterogeneous mutations of SH2D1A have been reported, although none are identical to that found in our patient. Deficiencies of this protein alter the function of NK and T cells and decrease cytokine production, subsequently affecting B cell proliferation and differentiation. This abnormal response renders the immune system unable to destroy cells infected with EBV [4]. 

SAP also appears to enhance apoptosis in B and T cells. Owing to the uncontrolled proliferation of T cells in SAP deficient patients, the majority of patients with XLP will progress to fulminant infectious mononucleosis with extensive hepatic necrosis and bone marrow failure leading to death within one month of onset of the disease. Survivors will typically exhibit residual cellular and humoral immunodeficiency, and are at increased risk for additional lymphoproliferative disorders and malignancies, usually of B cell origin [4].

XLP classically presents as fever, marked lymphadenopathy, malaise, pharyngitis, and hepatosplenomegaly. The diagnosis should be suspected in young males with an abnormal or exaggerated immune response to EBV, to include prolonged clinical course of greater than 1-2 weeks, marked cytopenias, viremia, or in those with a history of fatal EBV infection in maternal male relatives. Initial laboratory tests may reveal pancytopenia or lymphocytosis on the CBC, atypical lymphocytes on peripheral blood smear, positive Monospot or EBV titers, low fibrinogen, and elevation of bilirubin, triglycerides, and liver transaminases. Immunoglobulin levels can also be helpful, as hypogammaglobulinemia occurs in one-third of patients. Serial labs may demonstrate progressively worsening pancytopenias and liver function tests. Subsequent evaluation with neuroimaging, coagulation studies, and bone marrow biopsy can aid in determining the extent of disease. Definitive diagnosis is made by undergoing genetic testing for mutation in SH2D1A. 

XLP can have a similar clinical picture to hemophagocytic lymphohistocytosis (HLH) and in series of HLH patients mutations in SAP are frequently found. Thus, SAP mutation analyses should be considered in patients with HLH without apparent cause [5–7]. HLH can be familial in origin, or associated with various infections and autoimmune disorders. NK function and perforin studies can often be helpful in distinguishing these two entities; patients with HLH will exhibit decreased NK cell activity and perforin levels, while in XLP patients perforin levels are usually normal. A diagnosis of HLH is confirmed when analysis of bone marrow aspirate or lymph node tissue reveals hemophagocytosis [8]. 

The differential diagnosis of XLP also includes common variable immunodeficiency (CVID), which affects males and females equally and is characterized by frequent respiratory infections and low levels of IgG, IgA, and IgM [9]. Transient hypogammaglobulinemia of infancy can occur in infants without other complications and levels often normalize after 24 months of age. Griscelli syndrome is an autosomal recessive disease that features immunodeficiency, cytopenias, partial albinism, neurologic deficits, and viral induced HLH [10]. Chediak-Higashi is another autosomal recessive syndrome featuring partial albinism and immunodeficiency, but these patients also exhibit platelet dysfunction and can enter a lethal phase of disease initiated by viral infection (often EBV) leading to lymphohistiocytic infiltration of organs and death [11]. It is important to distinguish these entities from XLP as treatment approach and prognosis vary greatly. 

Unfortunately, individuals with XLP who enter the fulminant stage of disease have over 90% mortality, but some improvement has been shown with the HLH-94 protocol consisting of steroids and cytotoxic drugs such as cyclosporine and etoposide. Rituximab has reportedly been effective and IVIG has been used in the acute setting with minimal benefit. The only curative treatment for patients with XLP is an allogeneic bone marrow transplant, which ideally should be performed prior to EBV exposure. Thus, perinatal testing with chorionic villus sampling or amniocentesis is helpful in pregnancies of a known carrier [12].

Primary care physicians should be aware of XLP and suspect it in boys who develop complications with acute EBV infections. With suspected cases, hematology and immunology should be consulted for help in management and diagnosis. While treatment for affected children is still largely ineffective, it is essential to make a diagnosis so mothers who are carriers can receive appropriate genetic counseling and other family members may be screened.

##  Financial Disclosure/Proprietary Statement 

The authors have no financial, commercial, or proprietary interests in this paper and no affiliations/relationships to disclose.

## Figures and Tables

**Table 1:** Pertinent Lab Values.

		On Admission	After Treatment w/ antibiotics
White Blood Cell		3.9 K/*μ*L	
	Lymphs	68%	
	Atypical Lymphs	13%	
Hemoglobin		8.1 g/dL	
Platelets		36 K/*μ*L	

Bilirubin			8 mg/dL
ALT		146 *μ*/L	1890 *μ*/L
AST		152 *μ*/L	7300 *μ*/L
Triglycerides			319 mg/dL
Fibrinogen			107 mg/dL

LDH			2041 *μ*/L
